# Low socio-economic position is associated with poor social networks and social support: results from the Heinz Nixdorf Recall Study

**DOI:** 10.1186/1475-9276-7-13

**Published:** 2008-05-05

**Authors:** Simone Weyers, Nico Dragano, Susanne Möbus, Eva-Maria Beck, Andreas Stang, Stephan Möhlenkamp, Karl Heinz Jöckel, Raimund Erbel, Johannes Siegrist

**Affiliations:** 1 Department of Medical Sociology, University Duesseldorf, Heinrich-Heine-Universität Duesseldorf, Universitätsstrasse 1, Duesseldorf, Germany; 2 Institute for Medical Informatics, Biometry and Epidemiology, University Hospital, University of Duisburg-Essen, Essen, Germany; 3 West German Heart Center Essen, University Hospital, University of Duisburg-Essen, Essen, Germany; 4 Institute of Medical Epidemiology, Biometry and Informatics, Medical Faculty, Martin-Luther-University of Halle-Wittenberg, Halle, Germany

## Abstract

**Background:**

Social networks and social support are supposed to contribute to the development of unequal health within populations. However, little is known about their socio-economic distribution. In this study, we explore this distribution.

**Methods:**

This study analyses the association of two indicators of socio-economic position, education and income, with different measures of social networks and support. Cross-sectional data have been derived from the baseline examination of an epidemiological cohort study of 4.814 middle aged urban inhabitants in Germany (Heinz Nixdorf Recall Study). Bivariate and multivariate logistic regression analysis were carried out to estimate the risk of having poor social networks and support across socio-economic groups.

**Results:**

Socially disadvantaged persons more often report poor social networks and social support. In multivariate analyses, based on education, odds ratios range from 1.0 (highest education) to 4.9 (lowest education) in a graded way. Findings based on income show similar effects, ranging from 1.0 to 2.5. There is one exception: no association of SEP with close ties living nearby and regularly seen was observed.

**Conclusion:**

Poor social networks and low social support are more frequent among socio-economically disadvantaged people. To some extent, this finding varies according to the indicator chosen to measure these social constructs.

## Introduction

Social networks and social support have been recognized as important social determinants of health [1]. Social networks are supposed to affect health by shaping health-related norms and attitudes, by providing opportunities for social productivity or by acting as a psychosocial burden if lacking or conflicting. Social support is supposed to affect health by providing instrumental or emotional help to buffer stressful situations and their adverse health effects [2].

In the context of research on social inequalities in health in Western countries [3,4], social networks and social support are usually included as mediating or interacting variables. This might be justified, since their association with health has been documented, but few investigations only explored their social distribution. While it is generally assumed that social networks and social support are unevenly distributed, and "despite the very large and growing literature demonstrating the significance of social support for health and well-being, surprisingly little is known about the social distribution of this crucial resource" ([5], p. 193, [6]).

What does the evidence tell so far? There are study results that support the assumption of a *positive *association of socio-economic position with social networks and support, respectively. For instance, in the German Welfare Survey, the number of close ties increases with level of education [7]. Analyzing representative data from Western- and Eastern Germany, Andreß and colleagues found that in low income groups there are lower numbers of contacts to friends, and less satisfaction with social support [8]. Krause and Borawski-Clark conducted one of the few studies using different socio-economic indicators and different dimensions of social networks and support in a population of the elderly. They observed differences in contact with friends, support provided to others, and support satisfaction, with a higher prevalence in higher income and education groups [9]. Other studies, however, found an *inverse *association of socio-economic position and social networks and support, respectively. In a German study population, Knesebeck found less family contacts in higher socio-economic groups, whereas there was a mixed pattern with regard to receipt of and satisfaction with emotional support [10]. The MacArthur Study of Successful Aging found more ties and instrumental support in lower educational groups [11]. How do these contradictory findings fit together? It is argued that people living in poverty tend to have a more restricted relational 'radius'. In order to prevent social disadvantage and feelings of shame, they withdraw from larger society and have less friends but more ties to family, kinship and neighborhood [12].

One way of explaining these contradictory findings concerns variations in the choice of socio-economic indicators that can be associated with social networks and support in different ways. It is obvious that poverty can lead to social exclusion. Social participation requires a financial resource to afford activities, to pay member fees, to buy gifts etc. Therefore, it seems plausible that less external, non-family ties are established in low income groups. According to Bourdieu, economic capital can be transferred into social capital and vice versa [13]. Thus, accumulated relative deprivation among people with low social standing might be expected. In turn, better situated people dispose of more resources to provide support and, as a consequence, receive more of it. Yet, higher socio-economic position is not always associated with more or better social ties, for instance due to heavy work obligations or frequent absence from home. Given the contradictory body of research, we set out to explore the association of socio-economic position with social networks and support in a systematic way in the frame of a population based epidemiological investigation, the German Heinz Nixdorf Recall Study. Contrary to the selective use of these indicators in the above mentioned research we provide a comprehensive and generic measure of social networks and support (see methods). Equally, we introduce two indicators of socio-economic position to test respective associations.

## Methods

### Sample

Data were collected during the baseline examination of the German Heinz Nixdorf Recall (HNR) Study, an ongoing prospective population-based cohort study in Germany. The rationale, design and methods of this Study have been described in detail elsewhere [14,15]. The study base was the German population aged 45 to 74 years, living in three cities in an industrialized urban region (Ruhr Area). Participants were recruited from a random sample derived from mandatory citizen registries. 4814 men and women agreed to participate, which corresponds to a response proportion of 56% [15]. Comprehensive baseline examinations were conducted from December 2000 to August 2003. A five-year follow up is currently under way. The main aim of the HNR study is to improve prediction of coronary heart disease by combining established with new cardiovascular risk factors. Socio-economic position and social relations were included into the baseline screening as part of a psychosocial risk factor assessment through face-to-face interviews and paper and pencil questionnaires.

### Measures

#### Socioeconomic position (SEP)

*Education *was classified according to the International Standard Classification of Education as total years of formal education, combining school and vocational training [16]. The continuous variable was grouped into four categories, with the highest category indicating 18 and more years of education (equivalent to a University degree) and the lowest category indicating 10 and less years (equivalent to a basic school degree and no vocational training). *Income *was measured by equivalent household income including information on disposable income, household size and number of adults and children according to OECD criteria. For the present analyses income was divided into quintiles.

#### Social networks and support

Network measures include (1) availability of a confidant, (2) partnership, (3) close ties, (4) social participation and (5) a summary index of social integration. A *confidant *was defined as an intimate person to whom one feels close and whom one trusts. *Partnership *applied when respondents indicated to be married or to have a steady partner. *Close ties *were assessed by asking the total number of children, relatives and friends one feels close to. Based on this total number, we asked the number of those living close-by, those seen regularly and those having contact to at least several times per year. *Social Participation *in nine different groups and organizations was assessed. No participation in at least one of these group activities was defined as one risk of social isolation. The variables 'partnership', 'close ties' and 'participation' are combined into the *Social Integration Index *(SII) [17] which is categorized into four levels of integration ranging from social isolation (no partner, <= 2 total ties, no participation; level I) to high integration (partner, >= 12 ties, participation in two or more organizations; level IV). In two remaining levels, there are persons with a more or less favourable combination of partnership, ties and participation.

Support measures include *instrumental *and *emotional *support that were measured by a German adaptation of the New Haven EPESE questionnaire [18]. Questions first assess the availability of someone to help in daily tasks and the presence of one or more persons to approach when problems are experienced. In a second step it was asked who actually provided support and whether that support was appropriate. Based on the combination of this information, four categories were defined: 'support not needed', 'support appropriate', 'support inappropriate' and 'support needed but not available'. We defined lack of instrumental or emotional support if one of the latter two answers was given.

### Statistical analyses

The association of socio-economic position with social networks and support was tested in two ways: (a) mean values of close ties were compared across SEP categories by bivariate analyses; test for trend (p) is based on Jonckheere Terpstra test. Since we assume that age and gender interact with socio-economic position, we have also stratified our analyses accordingly in order to control respective confounding effects; (b) the indicators of poor social networks and support were regressed on education and income by multivariate logistic regression analysis. The reference category in these analyses was good social networks and support. Multivariate analyses were adjusted for age and sex. All statistical analyses were carried out using SPSS 12.0 programme.

## Results

Table 1 shows the distribution of the variables under study. Education is right-skewed with a higher prevalence of low educational degree. The mean income of 1566.00 Euros (707.30 SD) is below the Western-German average (1803.00 Euros in 2003).

**Table 1:** Distribution of variables

Characteristic (no. of missings)	Number [Mean]	% [SD]
Total sample	4814	100
Age (0)	[59.6]	[7.8]
Gender (0)		
Male	2395	49.8
Female	2419	50.2
Education – years of training (16)		
> 18	507	10.5
14–17	1068	22.2
11–13	2676	55.6
< 10	547	11.4
Household equivalent income quantiles (310)		
Min: 150,00; Max: 9500,00		
Percentiles		
20: 1833,3333 40: 2500,0000 60: 3166,6667 80: 4250,0000		
Confidant (39)		
yes	4157	87.1
no	618	12.9
Partnership (13)		
yes	4015	83.6
no	786	16.4
Close ties		
total number (48)	[10.3]	[7.2]
living nearby (78)	[7.3]	[6.0]
seen once per month (86)	[6.7]	[5.1]
contact phone/mail (77)	[8.0]	[6.7]
Participation (29)		
>= 1 group	2861	59.8
0 groups	1924	40.2
Social Integration Index (110)		
Level IV	217	4.6
Level III	1954	41.5
Level II	1960	41.7
Level I (Isolation)	573	12.2
Instrumental support (99)		
yes	4121	87.4
no	594	12.6
Emotional support (69)		
yes	3977	83.8
no	768	16.2

Almost one sixth of the study population reports to have no confidant and about one sixth reports to have no partner. The average number of close ties is about 10. A smaller proportion of these total ties only is available in everyday life, i.e. people living nearby (about 7) and seen at least once per month (about 7). This restriction of immediate exchange is compensated by telephone or mail (8). Four out of ten people do not join any group or organisation. Some 5 per cent are involved in a large social network as indicated by the Social Integration Index, but the majority indicates a medium-sized social network. However, 12.2 per cent are socially isolated. The majority of the population experiences appropriate social support. Yet, about one sixth belongs to a less privileged subgroup with lack of instrumental or emotional support.

### Socio-economic position and poor social networks/social support

Table 2 illustrates the association of socio-economic position with different indicators of social networks and support. In the respective left hand column, numbers (N) and percentages (%) of SEP groups reporting poor networks/support are represented; in the respective right hand column, odds ratios (OR) and 95% confidence intervals (CI) are represented, adjusted for age and sex. Lower SEP groups are compared to highest group.

**Table 2:** Socio-economic position and percentage/risk of poor social networks and support

	**No confidant**		**No partner**		**No participation**	
*Education*	*N (%) *^a^	*OR (CI) *^b^	*N (%)*	*OR (CI)*	*N (%)*	*OR (CI)*

1 (high)	28 (5.6%)	1.0	61 (12.0%)	1.0	153 (30.2%)	1.0
	125 (11.8%)	2.0 (1.3–3.0)	111 (10.4%)	0.9 (0.6–1.2)	361 (34.0%)	1.1 (0.9–1.4)
	354 (13.3%)	2.7 (1.8–4.1)	459 (17.2%)	1.0 (0.7–1.4)	1096 (41.2%)	1.6 (1.3–2.0)
4 (low)	111 (20.6%)	4.9 (3.1–7.7)	154 (28.2%)	1.4 (1.0–2.0)	306 (56.5%)	3.1 (2.4–4.0)
*Missing*	-		*1*		*8*	

*Income*						

1 (high)	68 (7.2%)	1.0	122 (12.8%)	1.0	333 (35.0%)	1.0
	67 (8.3%)	1.2 (0.8–1.7)	104 (12.8%)	0.9 (0.6–1.2)	285 (35.1%)	0.9 (0.8–1.2)
	133 (14.5%)	2.1 (1.5–2.8)	149 (16.2%)	1.2 (0.9–1.5)	364 (39.7%)	1.2 (1.0–1.4)
	153 (16.4%)	2.4 (1.8–3.3)	136 (14.5%)	1.0 (0.7–1.3)	384 (41.2%)	1.2 (1.0–1.5)
5 (low)	148 (16.9%)	2.5 (1.8–3.4)	239 (27.2%)	2.4 (1.8–3.1)	441 (50.4%)	1.8 (1.5–2.2)
*Missing*	*49*		*36*		*117*	

*Total*	*618*		*786*		*1924*	

	**Social Isolation SII Level I**		**Lack of instrumental support**		**Lack of emotional support**	

*Education*	*N (%)*	*OR (CI)*	*N (%)*	*OR (CI)*	*N (%)*	*OR (CI)*

1 (high)	49 (9.8%)	1.0	43 (8.6%)	1.0	58 (11.6%)	1.0
	92 (8.8%)	0.9 (0.6–1.3)	107 (10.2%)	1.2 (0.8–1.7)	160 (15.1%)	1.3 (0.9–1.8)
	319 (12.2%)	1.2 (0.8–1.6)	346 (13.1%)	1.4 (1.0–2.0)	424 (16.0%)	1.4 (1.0–1.9)
4 (low)	112 (21.0%)	2.1 (1.4–3.1)	98 (18.5%)	2.0 (1.3–3.0)	126 (23.6%)	2.3 (1.6–3.3)
*Missing*	*1*		-		-	

*Income*						

1 (high)	92 (9.7%)	1.0	77 (8.2%)	1.0	98 (10.3%)	1.0
	81 (10.1%)	1.0 (0.7–1.3)	86 (10.7%)	1.3 (0.9–1.8)	114 (14.1%)	1.4 (1.0–1.8)
	99 (11.0%)	1.1 (0.8–1.5)	116 (12.8%)	1.6 (1.1–2.1)	151 (16.5%)	1.7 (1.2–2.2)
	96 (10.4%)	1.0 (0.7–1.4)	123 (13.4%)	1.6 (1.2–2.2)	166 (18.0%)	1.8 (1.4–2.4)
5 (low)	180 (21.1%)	2.4 (1.8–3.2)	150 (17.4%)	2.2 (1.7–3.0)	186 (21.6%)	2.3 (1.8–3.0)
*Missing*	*25*		*42*		*53*	

*Total*	*573*		*594*		*768*	

^a^) Number and percentage; ^b^) Odds Ratio (95% Confidence Interval); model adjusted for age and sex;

Findings show that socially disadvantaged persons are more often exposed to poor social networks and social support. In bivariate analyses it becomes obvious that there is a higher percentage of having no confidant and no partner, of no participation, of being socially isolated and of lacking social support. In multivariate analyses, odds ratios are elevated in low SEP groups. Based on education, odds ratios range from 1.0 (highest education) to 4.9 (lowest education). Findings based on income show similar effects, ranging from 1.0 to 2.5. In some cases (no confidant, poor social support), the association of SEP with poor social networks and support gets stronger with decreasing SEP, but by far the strongest effects are observed in most disadvantaged groups.

Structural measures (SII) show effects comparable with qualitative support measures. If we decompose the Social Integration Index, we see that having no confidant shows the strongest effect in both SEP groups; the lowest effect was found for partnership in education and for participation in income groups.

### Socio-economic position and close ties

**Figure 1:**
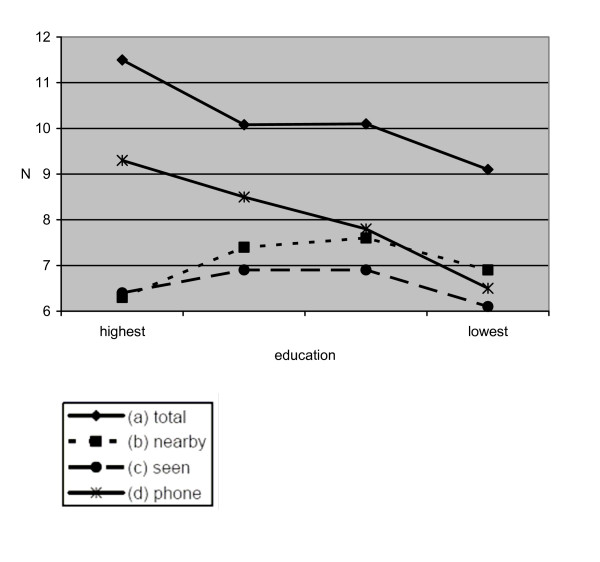
Socio-economic position (education) and close ties (mean values).

**Figure 2:**
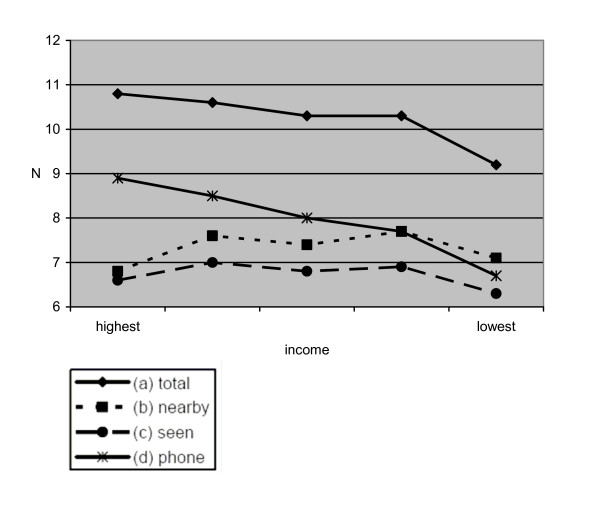
Socio-economic position (income) and close ties (mean values)  Legend

Figures 1 and 2 depict the mean values of close ties compared across SEP categories education and income. Figure 1 shows that the total number of close ties (a) significantly diminishes with decreasing educational status (p for trend = .000). Concerning the number of those living close-by (b) no such relationship is observed. Here, we find an insignificant u-shaped association (p for trend = .105). As regards those regularly seen (c) we find the same u-shaped association slightly below significance level (p for trend = .047). It is of interest to note again a positive relationship of educational status with close ties via communication media (d; p for trend = .000). The same pattern is observed for the second socio-economic indicator income (figure 2). To rule out possible age and gender effects, we stratified the analyses accordingly and results remained basically unchanged (results not shown).

It becomes obvious that there is a difference between the number of reported ties and the number of ties with persons who are actually seen. Another interesting finding is that this difference widens with increasing socio-economic position.

At the methodological level it should be mentioned that standard deviations of means are rather large. Therefore, we repeated our analyses using the median number of close ties. Again, results remain basically unchanged.

## Discussion

This study provides a systematic test of associations of SEP with social networks and support using a range of indicators. Mostly, we observe that low status people are at increased risk of being structurally isolated and of receiving inappropriate support. They report lower numbers of close ties, have a higher risk of having no confidant and no partner, of not participating in any club, in other words of being socially isolated (table 2). Also, they report more often to lack instrumental and social support. This finding fits to Bourdieu's theory of an accumulated relative deprivation among people with low social standing (see above).

However, with regard to the availability of social ties, i.e. number of close persons living nearby and seen at least once per month, we found no such relationship (figure 1). Numbers are more or less equally distributed across SEP groups. This is in line with an alternative argument mentioned in the introduction that higher socio-economic position is not always associated with more social ties, for instance due to heavy work obligations or frequent absence from home which might hamper the development of stable relations. As regards the socio-economic indicators, we do not find a clear pattern of which indicator represents a higher risk of poor social relations. Taken together, whether there is an association of socio-economic position with social networks and support partly depends on the indicator of the latter factors.

Given this evidence, it seems justified to conclude that social networks and support are included into the set of variables associated with social inequalities in health. Yet, attention needs to be given to the way of operationalizing these latent constructs.

### Strengths of the study

A particular strength of the study is given by the fact that it is based on a large community sample rather than on a specific population such as an occupational cohort. Furthermore, within our sample we controlled for sample bias in terms of status-specific refusal rates. In fact, participation rates were lower among lower SEP people, but with the help of an additional subgroup analysis of non-responders we were able to identify the observed association with low social participation in this non-responder group as well [15].

Another strength concerns the measurement of variables of social relationships. Instead of using non-systematic or proximate indicators we applied original validated questionnaires assessing a comprehensive range of indicators of social relationships with internationally comparable items [17-19]. The Heinz-Nixdorf-Recall-Study has been externally certified, thus confirming a high quality of data collection and data handling.

### Limitations

These strengths are balanced by several methodological limitations. First, we cannot rule out a social desirability effect that may weaken the validity of some indicators, such as reported household income [20,21], or the assessment of close social relationships. Moreover, the concept of 'friendship' may be more familiar to middle class compared to lower class people [22,23] although we provided a clear description of the concept during the interview.

A second limitation concerns our choice of socio-economic indicators. Since only one third of the study population was working at the time of the interview, we excluded occupational status as an additional SEP indicator. In a subgroup analysis where occupation was classified according to ISCO-88 and, additionally, according to the Treiman-prestige-scale [24], a less consistent pattern was observed with regard to social networks and support. Low occupational status was significantly associated with having no confident and with lack of participation, but was unrelated to the remaining measures of social relationships. Moreover, important sociodemographic factors such as age and gender were analysed as confounders rather than variables subjected to indepth-subgroup analysis. Future studies should complement the current approach.

Finally, in the cross-sectional study, no information on the temporal relation between socio-economic position and social networks/support was available. While we hypothesize that socio-economic position to some extent determines the range and quality of social relationships, we cannot exclude a reverse association. The prospective design of this study will allow us to test the direction of associations in future analyses.

### Implications

This rather consistent association of SEP with social relations is supported by another German study which recently identified 8 per cent of the German population as living and working under precarious conditions with accumulated material and psychosocial disadvantage [25]. Interventions to tackle social isolation should be carried out in disadvantaged settings and in highly vulnerable groups, such as elementary schools or deprived urban settings, and in unemployed people or lone mothers. Some instructive examples can be found in [26].

## Conclusion

Poor social networks and low social support are more frequent among socio-economically disadvantaged people. To some extent, this finding varies according to the indicator chosen to measure these social constructs. Nevertheless, results underline the need of developing interventions to improve support and extend networks among low SEP groups.

## Competing interests

The authors declare that they have no competing interests.

## Authors' contributions

KHJ, RE, JS are principal investigators; SM, AS were responsible for the study coordination; ND, JS developed the medical sociological examination; EMB lead the study examinations; SW performed the statistical analysis of the present paper. All authors read and approved the final manuscript.
